# Implementing an outreaching, preference-led stepped care intervention programme to reduce late life depressive symptoms: results of a mixed-methods study

**DOI:** 10.1186/s13012-014-0107-y

**Published:** 2014-08-28

**Authors:** Ilse MJ van Beljouw, Miranda GH Laurant, Marjolijn Heerings, Max L Stek, Harm WJ van Marwijk, Eric van Exel

**Affiliations:** Department of Psychiatry, VU University Medical Center/GGZ inGeest and EMGO+ Institute for Health and Care Research, A.J. Ernststraat 1187, 1081 HL Amsterdam, The Netherlands; Radboud University Medical Center, Scientific Institute for Quality of Healthcare (IQ healthcare), 114-IQ healthcare, PO Box 9101, 6500 HB Nijmegen, The Netherlands; HAN University of Applied Sciences, Faculty of Health and Social Studies, PO Box 6960, 6503 GL Nijmegen, The Netherlands; Department of Psychiatry, VU University Medical Center/GGZ inGeest and EMGO + Institute for Health and Care Research, Amstelveenseweg 589, 1081 JC Amsterdam, The Netherlands; Department of General Practice and Elderly Care Medicine, VU University Medical Center and EMGO + Institute for Health and Care Research, van der Boechorststraat, 1081 BT Amsterdam, The Netherlands

**Keywords:** Aged, Depression, Mass screening, Health knowledge, Attitudes, Practice, Patient acceptance of healthcare, Qualitative research, General practice, Elderly

## Abstract

**Background:**

Depressive symptoms are highly prevalent in old age, but they remain mostly untreated. Several clinical trials have shown promising results in preventing or reducing depressive symptoms. However, it is not clear how robust these effects are in the real world of day-to-day care. Therefore, we have implemented the ‘Lust for Life’ programme, which significantly reduced depressive symptoms in community-dwelling older adults in the first three months after implementation. This mixed-methods study was conducted alongside the trial to develop a contextualised understanding of factors affecting the implementation.

**Methods:**

A total of 263 persons of 65 years and older with depressive symptoms were recruited from 18 general practices and home care organizations in the Netherlands. We used qualitative data (in-depth interviews and focus group discussions with participants with depressive symptoms and healthcare professionals) as well as quantitative data (longitudinal data on the severity of depressive symptoms) to explore hindering and facilitating factors to the implementation of the ‘Lust for Life’ programme.

**Results:**

The uptake of the routine screening was poor and imposed significant burdens on participants and healthcare professionals, and drop-out rates were high. Participants’ perceived mental problems and need for care played a key role in their decision to participate in the programme and to step up to consequent interventions. Older people preferred interventions that focused on interpersonal contact. The programme was only effective when delivered by mental healthcare nurses, compared to home care nurses with limited experience in providing mental healthcare.

**Conclusions:**

The intervention programme was effective in reducing depressive symptoms, and valuable lessons can be learned from this implementation trial. Given the low uptake and high investment, we advise against routine screening for depressive symptoms in general healthcare. Further, agreement between the participant and healthcare professional on perceived need for care and intervention is vital. Rather than providing a stepped care intervention programme, we showed that offering only one single preference-led intervention is effective. Lastly, since the provision of the interventions seems to ask for specific skills and experiences, it might require mental healthcare nurses to offer the programme.

**Trial registration:**

Dutch trial register NTR2241

**Electronic supplementary material:**

The online version of this article (doi:10.1186/s13012-014-0107-y) contains supplementary material, which is available to authorized users.

## Background

Depressive disorders are the second leading cause of disability worldwide [1]. Although many efficacious interventions are available, most depressive disorders remain untreated, particularly in older adults [2,3]. Therefore, several strategies have been proposed to improve depression management, aimed at treating as well as preventing onset and recurrence [4].

First, one of those strategies concerns mass screening in general practice to improve case findings. Screening aims to detect persons with depressive symptoms at risk for major depression, or those with a depressive disorder that has remained unrecognised by healthcare professionals.

Second, to improve the provision of effective (preventive) interventions, stepped care models, and preference-led care have been developed. Stepped care aims at making the best use of available resources by initially offering effective interventions at the lowest possible intensity (*e.g*., guided self-help), which are advanced to a higher intensity when needed (*e.g*., counselling, medication). Stepped care is guideline-concordant and is effective in preventing and treating depressive disorders [5-11]. Treatment initiation and adherence could further be improved by applying preference-led care [12], *i.e*. tailoring treatment to participants’ needs by providing them in each step with multiple treatment options to choose from [7,13-15]. Yet, current randomised clinical trials have compared stepped care models to stratified care, and thereby missed participants’ needs for care.

**Figure 1:**
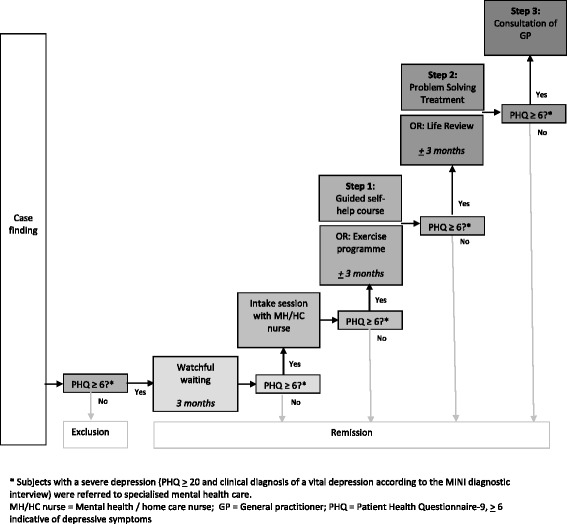
**Treatment algorithm.**

It is unknown how a preference-led model – integrated with the provision of stepped care – would impact clinical effectiveness, or be effective when implemented in routine primary care. Therefore, we implemented an intervention programme (the ‘Lust for Life-programme’) consisting of evidence-based interventions to reduce depressive symptoms in older adults in primary care and home care facilities in the Netherlands. This programme consisted of two key aspects: first, it was outreaching, *i.e*., we applied a pro-active case-finding procedure, and second, both stepped and preference-led care was provided by offering multiple treatment options in steps (see Figure 1). The current mixed-method study was conducted alongside, to develop a contextualised understanding of factors affecting implementation.

## Methods

### Study design

The ‘Lust for Life’ programme was implemented in 18 general practices on three different sites (Amsterdam, West-Friesland and Leiden) in the Netherlands and one home-care facility located in Amsterdam. These facilities were randomised into four clusters following a stepped-wedge randomised-cluster design [16], see Additional file 1. A stepped-wedge design is a type of crossover design in which the intervention is rolled out sequentially to participants over a number of time periods. All participants are recruited at the start of the study and assigned to several clusters. Starting moments are determined by (cluster-) randomisation and by the end of the study, all participants will have received the intervention. That is, clusters cross over from control to intervention condition. During the control condition, usual care is provided. This design was chosen since the offered clinical interventions were evidence-based, and it might, therefore, be unethical to withhold them from a considerable group of subjects as would occur in a randomised clinical trial. Second, staged implementation allowed for the evaluation, refinement and application of the (renewed) implementation strategy along the way. The VU University Medical Centre Ethical Review Board approved of the study (No. 2010/084).

### The ‘Lust for Life’ programme

Participants were recruited by proactive case finding to lower barriers to care utilisation. All enlisted persons of 65 years and older (N = 9,661) from participating general practices and a home care organisation were informed about the programme and invited to fill out the self-report Patient Health Questionnaire-9 (PHQ-9; [17]), which is a dual-purpose instrument to diagnose a depressive disorder, as well as monitor depression severity.

Eligible subjects who gave informed consent were offered three months of watchful waiting, and consequently invited for an intake session when depressive symptoms persisted (Figure 1). The stepped care programme consisted of preference-led, evidence-based interventions administered in three steps, if necessary: step 1: choice of 1a) a guided self-help course based on Lewinsohn’s ‘Coping with Depression Course’ [18] or 1b) an exercise programme [19] delivered in groups of four to six participants three times a week; step 2: choice of 2a) life review (a structured reminiscence intervention aimed at reducing late life depressive symptoms) [20] or 2b) Problem Solving Treatment (PST; structured skills enhancing behavioural intervention based on the assumption that problems in daily life cause and maintain depressive symptoms [21]); and step 3: referral to a GP to discuss further treatment options. Nurses were provided with instructions on how to refer older people with feelings of loneliness to social services, if desirable.

The clinical interventions were offered to participants with continuous depressive symptoms according to the cut-off score of six or higher [22] on the PHQ-9. Patients with a PHQ ≥20 (severe depression) and vital depression on diagnostic interview (MINI [23]) were advised to consult their GP immediately.

The intake session and most of the clinical interventions were provided by mental healthcare nurses (to participants recruited in general practices) or by home care nurses (to participants recruited in general practices or home care). Geriatric physiotherapists offered the training programme. A psychologist specifically trained nurses on how to deliver the clinical interventions. They attended two-monthly group supervision meetings and received individual feedback on at least two tape-recorded sessions of the step two clinical interventions. Personal supervision by telephone was provided upon request. A qualified psychologist supervised. Except for the exercise programme that took place in a gym, interventions were administered individually at the participants’ homes or in the practice of the participating GP.

### Clinical effectiveness of the programme

Intention-to-treat-analyses showed that the ‘Lust for Life’ programme had a favourable impact on depression severity: depressive symptoms significantly declined in the first three months after implementation of the clinical interventions compared to their course before implementation (Wald = 19.636, df = 8, p = 0.012). Estimated means decremented with 1.5 points on the PHQ-9 from an average of 9.34 (95% CI 8.13 to 10.54) to an average of 7.83 (95% CI 6.83-8.82) [24]. This effect can be compared to a standard mean difference of 0.25 (SD 6.0) or a small effect as used by the Cochrane Collaboration.

### Data collection

#### Qualitative data collection

**Figure 2:**
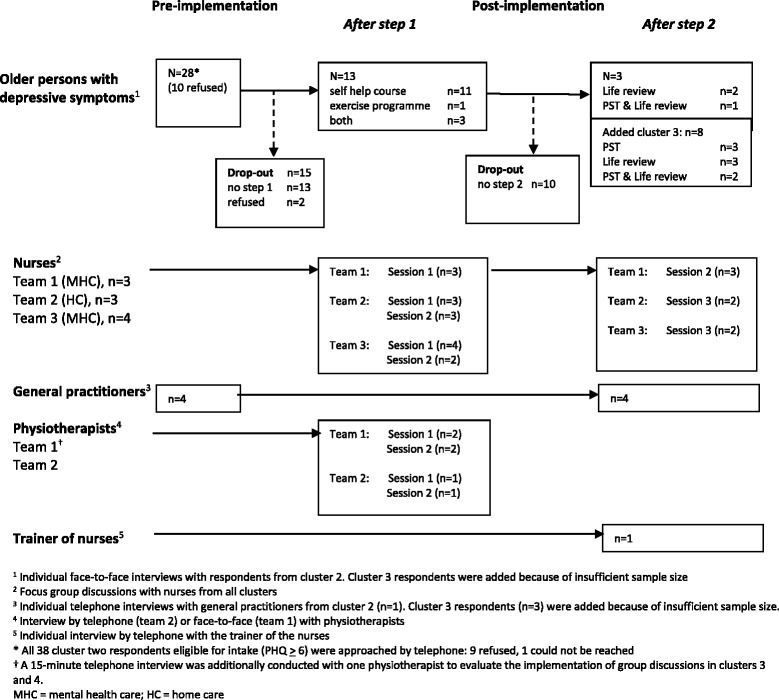
**Overview of qualitative data collection: interviews and focus group discussions with different stakeholders.**

The main sources for identifying, facilitating and hindering factors to the implementation of the ‘Lust for Life’ programme were qualitative interviews with different stakeholders at several moments during the implementation of the intervention programme (see Figure 2 for more detailed information). Individual interviews were conducted with respondents with depressive symptoms. All cluster-two respondents were invited to participate in the interviews and were followed up on, irrespective of their decision to accept or refuse the intervention offer and possible drop-out of the interventions, GPs, physiotherapists and the trainer of the interventions. Focus group discussions were held with teams of mental healthcare and home care nurses.

Since it was impossible to interview non-respondents or older adults who declined participation, we collected data in other ways to capture their views. Information was used from returned screening forms and telephone conversations that research staff conducted with elderly people. We also used ‘proxies’ by gathering information from healthcare providers on behalf of the elderly people about non-participation.

One of two interviewers (MH or IvB) held individual interviews face-to-face at respondents’ homes (participants) or by telephone (GPs, physiotherapists, and trainer). MH led focus group discussions at subjects’ working places. They lasted between 5 and 87 minutes (participants), 39 and 96 minutes (team of nurses), 6 and 22 minutes (individual GPs), 32 and 51 minutes (physiotherapists), and 43 minutes (trainer). All interviews and focus group discussions were digitally recorded with the consent of the participants, and field notes were made. Data were collected between September 2011 and June 2012.

### Quantitative data collection

The process evaluation used qualitative and quantitative data to triangulate significant findings [25]. Data from participants with depressive symptoms were collected at baseline (telephone interviews and self-report questionnaires) and every three months (self-report questionnaires). Additionally, nurses were requested to fill out evaluation forms for each client about the course of the treatment.

### Measurements

#### Qualitative measurements

Semi-structured interview guides were created for each stakeholder that consisted of open-ended questions and were parallel in content as much as possible. To enquire about all relevant factors enabling or hindering the implementation process, Grol and Wensing’s framework was used to build the interview guides [26]. This framework was derived from various theories and models on determinants of implementation practice and included the following levels: factors related to the innovation; individual professional; patient; and context (social, organisational, economic and political context). As we believe that factors may differ across the different phases of the implementation process, these levels were studied pre- and post-implementation by interviewing respondents at different moments during the implementation process.

Stakeholders were asked about their expectations for the ‘Lust for Life’ programme, motivation for participation, experiences with various components of the programme and strategy (*e.g*., case finding, [transition to] interventions, prerequisites for optimal implementation). The main focus was on factors that facilitate or hinder implementation. Interview guides also contained stakeholder-specific questions. In the interviews/focus group discussions with nurses and physiotherapists, certain important themes emerging in interviews in one group of stakeholders were verified with participants in another member group in subsequent interviews when they did not come up spontaneously.

### Quantitative measurements

Severity of depression, the primary outcome measure for the quantitative analyses, was measured with the PHQ-9, which consists of nine items with total scores ranging from 0 to 27. Subjective need for care was addressed at baseline by the Perceived Need for Care Questionnaire (PNCQ; [27]). The PNCQ is a fully structured interview that assesses the subject’s perception of the presence of a mental problem, the perceived need for treatment, and the use of healthcare services in the past three months. Subjects who received care for a mental problem were asked if their needs were (fully) met. Confounders included educational level, age, dwelling place, and physical functioning (Modified Katz Activities of Daily Living) (KATZ ADL; [28]), at baseline.

### Data analysis

Initially, qualitative and quantitative data were analysed separately. Results on the same topic were integrated later to verify and corroborate findings from different approaches about the same phenomenon (triangulation; [29]) and to elaborate or clarify the results from one method by the other (complementarity; [30]).

### Qualitative data analysis

All interviews and focus group discussions were transcribed verbatim. Transcripts were read, and reread, and coding trees consisting of key themes and subthemes that emerged from the data were built in Atlas 5.2 for each stakeholder. For instance, the coding tree for the focus group discussions with nurses consisted of several key themes such as ‘guided self-help: experiences from nurses’. Within the key themes, different levels of codes were constructed and ordered within ‘facilitating factors’ or ‘hindering factors’ to the implementation of the intervention programme (*e.g*., ‘hindering factor - the level of guided self-help course’). Each code further consisted of several subcodes (*e.g*., ‘guided self-help course is too difficult for self-study,’ ‘guided self-help course is too confronting,’ etc.).

MH and IvB coded transcripts independently, and discussed until they reached consensus. To ensure that data were understood from different perspectives, an experienced researcher in implementation research (ML) was closely involved in data coding and interpretation. Coded interviews were scrutinized for underlying themes and associations between themes by applying a grounded theory approach [31]. Data were further examined for convergent or divergent perspectives from different stakeholders. Summaries of (sub)codes were written and exemplified by adding quotations from the original transcripts.

Data on reasons for declining the intervention offer provided by non-respondents on the screening questionnaire or during the telephone conversation with research staff were coded separately and consequently compared to data derived from the interviews and focus group discussions.

Peer debriefing took place by regular team meetings in which original data, summaries of (sub)codes and results were presented and discussed [32]. Finally, all findings and codes were additionally clustered according to the two fundamental aspects of the programme: case finding, and the stepped care preference-led interventions (see Table 1).

**Table 1:** **Overview of determinants that facilitated or hindered the implementation of the ‘Lust for Life’ programme**

**Theme**	**Level**	**Factor**	
I. Proactive case finding	Participants with depressive symptoms	*Illness perceptions*	Depressive symptoms were seen as normal ageing and not perceived as burdensome.
		*Perceived need*	The usefulness of the programme was questioned, or people preferred to handle problems themselves.
	Healthcare professionals (and their interactions)	*Attitude toward screening*	Case finding was the main reason for GPs to participate; nurses were more critical.
	Innovation (*i.e.,* case finding procedure)	*Experiences with case finding*	Included participants had no problem with being screened. However, screening was problematic for the entire patient population in a general practice.
		*Perceived results of case finding*	Were limited, attracted persons from non-target groups, and were perceived to not reach a considerable part of the target group.
	Context	*Availability of treatments*	Other treatments for this target group were already available.
II. Personalised, stepped care clinical interventions	Participants with depressive symptoms	*Preferences for the interventions*	Choices for the exercise programme and Life Review were made more easily and with more enthusiasm compared to the other interventions.
		*Transitions to subsequent steps*	Were limited, and mostly determined by participants’ illness perceptions.
	Healthcare professionals (and their interactions)	*Professional backgrounds*	Home care nurses felt insufficiently equipped to provide the interventions, questioned their effectivity and the eligibility of participants for the study. Mental healthcare nurses were confident of their own skills and perceived participants’ limited motivation as a challenge to their jobs.
		*Professional interactions*	Nurse and physiotherapists did not perceive working as a team, and missed out on information and limited involvement from one another.
	Innovation (*i.e*., the clinical interventions)	*Intervention choice and effectiveness*	The course of depressive symptoms was similar for people who participated in the various interventions. Drop-out was higher in persons who participated in PST than in life review.
		*Suitability of the interventions*	Participants highly valued their interaction with the nurses/ physiotherapists. The exercise programme and Life Review were perceived as meeting participants’ needs; many hindering factors were mentioned about the self-help course.
**Theme**	**Level**	**Factor**	
		*Provision of the interventions*	• Much guidance was required in the provision of the self-help course.
			• Physiotherapists missed opportunities to discuss depressive symptoms with participants; the exercise programme could not be provided according to the protocol’s demanded intensity.
			• Nurses questioned whether participants adopted the intervention methods in Life Review and PST.
			• Referrals to specialised mental healthcare when depressive symptoms remained were not always considered appropriate.
	Context	*Embedment in different organisations*	Limited embedment caused high work pressure on the home care nurses. Embedment of the programme in specialised mental healthcare facilitated additional treatment by this organisation when depressive symptoms remained.

### Quantitative data analysis

Data were analysed according to the intention to treat principle using Generalized Estimating Equations with an autoregressive correlation structure to take into account repeated measurements within patients. Time trend was corrected for by including the time since the start of the first implementation (in units of three-month intervals) as a categorical variable in all models. The start of implementation was individually defined for each subject as the date of their intake session with the nurse. In the subgroup analyses, nurses’ backgrounds, and intervention decisions made were separately added to the model as interaction terms, corrected for confounders (age, educational level, the dwelling place, and daily functioning).

## Results

Table 1 presents an overview of the relevant themes and underlying levels relevant to the implementation of the ‘Lust for Life’ programme. Quotes are from participants, home care (HC) or mental healthcare (MHC) nurses, GP and the trainer.

### Theme I: proactive case finding

**Figure 3:**
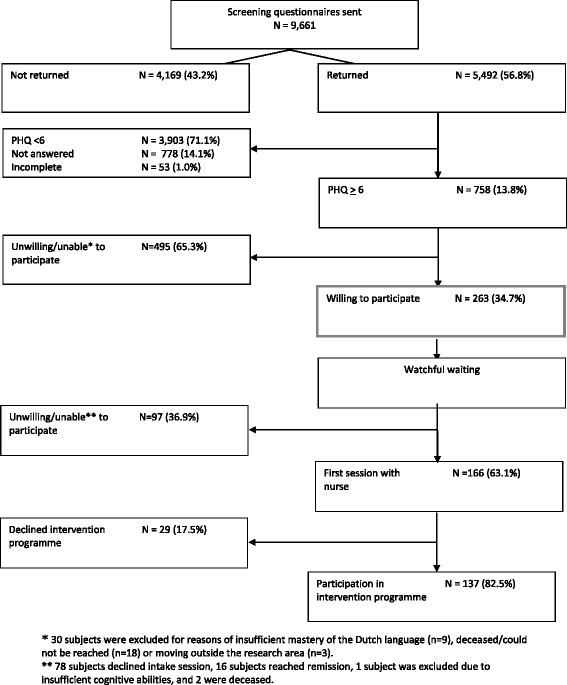
**Case finding results.**

The first essential aspect of the ‘Lust for Life’ programme examined was pro-active case finding. All enlisted persons of 65 years and older (N = 9,661) from 18 general practices and one home care facility were screened for depressive symptoms using the self-report PHQ-9. Figure 3 shows that 263 persons wanted to participate and met inclusion criteria, and 138 enrolled (52.5%).

Since the prevalence of depressive symptoms in community-dwelling older adults is estimated at 13.5%, the case-finding procedure was aimed at the approximately 1,300 persons with depressive symptoms among all those 9,661 invited. Eventually, we only included 2.7% of all screened individuals in the study and 1.5% took part. The semi-structured interviews and focus group discussions with all stakeholders provide an understanding of this limited reach. Notably, perspectives from respondents with depressive symptoms were presented by those who were included, and consequently decided to accept or refuse our intervention offer, unless specified otherwise.

### Factors related to participants with depressive symptoms

Two factors emerged at the level of older people with depressive symptoms: participants’ illness perceptions, and their perceived needs for the ‘Lust for Life’ programme.

### Illness perceptions

The way older people with depressive symptoms (*i.e*., PHQ ≥6) perceived their distress appeared to have great influence on their willingness to participate. Quantitative material showed that among all participants who screened positive for depressive symptoms (N = 758), many stated not feeling ‘down, depressed or hopeless’ (PHQ, N = 172 [28.1%]). Many declined participation for this reason: 69 persons (21.5% of those who provided reasons for refusal on the screening questionnaire) indicated that they did not perceive depressive symptoms. Also, several participants who were included in the study did not perceive ‘any mental or emotional problems’ (PNCQ, N = 74 [28.1%]).

Qualitative results confirmed this finding: Approximately half of all subjects did not feel that (medical) terms such as having a depressed mood or being depressed were applicable to their situation, especially those who declined the intervention offer. Moreover, emotional distress was often not experienced as burdensome but as part of normal ageing, in particular among those who declined the interventions (see Additional file 2: Box 1, quote 1).

Next, the qualitative interviews shed light on self-perceived causes of emotional distress. For most participants, their perceptions differed significantly from the biomedical model that presents depressive symptoms as a medical illness. Rather, ageing-related losses such as declining health and loneliness were perceived as the most prominent problems that caused mood disturbances.

### Perceived need for the ‘Lust for Life’ programme

Older people’s attitudes toward the programme were a second important factor that contributed to low use of care. In our quantitative data, we found that almost half of all participants with depressive symptoms stated that they did not perceive a need for care for a mental problem on the PNCQ (39.5%, N = 104) or that their need for care was already met (8.0%, N = 21). Among those with an unmet need for care (49.8%, N = 131), preferring to handle emotional problems themselves was the most frequently mentioned reason for this need to be unmet (31.5%, N = 134 [reasons could be provided for multiple needs to be unmet]). Most persons not included to the study with depressive symptoms (PHQ ≥6) who provided reasons for their refusal on the screening instrument stated that they perceived no need for care (27.4%, N = 88).

The same findings emerged from the qualitative data. Among those who perceived depressive symptoms as hindering, many questioned whether they would deserve to receive the intervention. For instance, because they preferred to manage problems themselves, or they perceived their problems as a status-quo, that cannot or does not have to be changed (see Additional file 2: Box 1, quote 2). This appropriateness issue was also recognised by the healthcare professionals, as stated by a mental healthcare nurse (see Additional file 2: Box 1, quote 3). During the focus group interviews, nurses also mentioned that many participants already had sufficient coping skills to handle their problems themselves.

### Factors related to healthcare professionals (and their interactions)

Case finding was the main aim for GPs to participate in the study. They wished to get more insight into the mental wellbeing of their patient population. Nurses also valued the aim of lowering barriers to care for people who are reluctant to ask for help. However, their attitudes toward screening were more critical, in particular of the nurses of a mental healthcare organisation that had previously participated in a research project using proactive case finding to include participants [8]. Nurses felt that proactive case finding failed to include the target group of people with depressive symptoms, who would benefit from the interventions for several reasons. First, persons with a depressed mood who had little insight into their emotional problems would be less inclined to fill in the screening questionnaire compared to people who are more self-reflexive (see Additional file 2: Box 2, quote 4). Second, they were concerned that the procedure might have a ‘honeypot effect’ on persons with particular personality-related problems who would not benefit from the interventions. Third, a screening procedure was considered to be too insensitive to the fluctuation of people’s moods and would, therefore, lead to the unwanted exclusion of many eligible persons.

### Factors related to the innovation

Two factors emerged within the level of the innovation (*i.e*., the case finding method): experiences with the case finding procedure, and their perceived results.

### Experiences with the case finding procedure

Most members had no problems with being screened for depressive symptoms or even appreciated their GP’s consideration. However, GPs found that the system caused considerable disturbance among their entire more elderly patient population. They suggested that several elderly people were upset when they received the screening questionnaire (see Additional file 2: Box 3, quote 5). GPs also noticed that the screening procedure was stressful to their patients since many older adults did not know how to respond to the invitation. Perhaps this was caused by our finding that many older people did not have any idea what the programme entailed, despite our effort to carefully provide information about the programme by mail and telephone. The research team also noticed this during conversations with respondents who declined participation in the study. Subsequently, it was cited by nurses and most of the elderly people who took part in the interviews. However, many participants did not express the need for more information (see Additional file 2: Box 3, quote 6).

### Perceived results of the case finding procedure

Although GPs and nurses agreed with the inclusion of most people, they mentioned that the outcomes in numbers of persons taking part in the clinical interventions were disappointing. They stated furthermore that the system also attracted persons from non-target groups such as individuals with personality disorders. Screening did not provide information on whether participants perceived a need for care, or whether a considerable part of the target group was not reached (see Additional file 2: Box 3, quote 7). On the other hand, nurses and GPs appreciated that the case finding system facilitated the inclusion of respondents who had not yet expressed a need for care to their physician.

### Context-related factors

Few context-related factors were mentioned of influence on the low uptake of the case-finding procedure. Only GPs stated that various treatments were already available for this group of older people with depressive symptoms and, therefore, the programme did not offer anything new.

### Theme II: the preference-led, stepped care interventions

**Figure 4:**
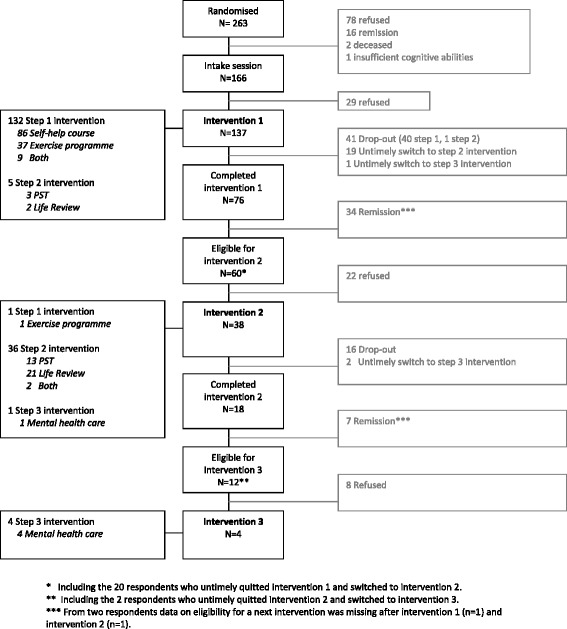
**Participants’ course during the intervention programme.**

To facilitate understanding of the factors related to the clinical interventions that were of influence on the implementation of the ‘Lust for Life’ program, Figure 4 shows the older adults’ treatment courses throughout the programme.

Treatment courses could vary for individuals in clusters one and two, where people could only begin with step one intervention. In clusters three and four, people were given the opportunity immediately to start with step two interventions if desirable, and drop-out rates are given for the differing clusters (data not shown in Figure 4). In clusters one and two (N = 137), 47 persons (34%) fell out of the programme after the intake session (N = 20, 14%), or quit the guided self-help course or the exercise programme untimely (and dropped-out of the program; N = 27, 20%). In clusters three and four (N = 126), participants could start immediately with step two interventions: 22 persons (17%) dropped out during the first period of the implementation process: 9 persons (7%) dropped out after the intake session, and 13 individuals (10%) quit the intervention of their choice untimely (and dropped out of the program). In clusters three and four, only 5 persons (4%) decided immediately to start with step two interventions.

### Factors related to the participants with depressive symptoms

Two factors emerged at this level: participants’ preferences for the clinical interventions and the transition to subsequent steps in the programme.

### Participants’ preferences for the clinical interventions

Of all persons who took part in the interventions (N = 137), most chose to participate in the guided self-help course (N = 86, 63%; Figure 4). A total of 38 persons (28%) signed up for the exercise programme, and 9 individuals participated in both (7%). Among elderly people who followed a step two intervention, life review was more frequently chosen (N = 23, 56%) than PST (N = 16, 39%); two individuals participated in both interventions (5%).

It became clear that older adults’ illness perceptions played a crucial role in the treatment decisions they made. Interestingly, we noticed that persons’ choices for the training programme and life review were more natural and more convincingly made than for other interventions (see Additional file 2: Box 4, quote 8). Perhaps their content was easier to capture, which facilitated decision making, or these interventions were considered less stigmatising since they less stressed their aim of alleviating mental problems. When motivating their choice for the training programme or life review, more aged people mentioned reasons that were hardly related to reducing mental distress, such as enjoying sports or loving to talk about the past (see Additional file 2: Box 4, quote 9).

Reasons for participants to participate in the guided self-help course were that they preferred to work on the intervention in their own time or because they wanted to give the self-help book the benefit of the doubt. For several elderly people in clusters one and two, it was the second best option since they already exercised, were too limited in their mobility to reach the gym, or did not like to join a group activity. None of the older people who participated in PST could provide an explanation for why they had chosen this intervention.

### Transitions to subsequent steps

Older adults with persistent depressive symptoms (PHQ ≥6) were offered participation in a subsequent step after having finished their first intervention. Of those eligible (N = 60), 38 (57%) persons took part in the second intervention, and 4 out of 12 eligible (33%) in a third (referral to the GP). A total of 19 persons (7%) were offered an early switch to the second intervention to prevent drop-out.

Again, illness perceptions were essential to persons’ decisions to take part in additional interventions. Few elderly people mentioned that they perceived persistent depressive symptoms that motivated them to accept further treatment (see Additional file 2: Box 4, quote 10). For many other persons, however, their lack of self-perceived depressive symptoms or need for help were decisive to refuse further treatment in step two (see Additional file 2: Box 4, quote 11).

### Factors related to the healthcare professionals (and their interactions)

#### Relationship with the effectiveness of the ‘Lust for Life’ programme

Quantitative analyses revealed that the effectiveness of the clinical interventions varied with the kind of nurses who provided the interventions (Wald = 26.659, df = 8, p = 0.001). Stratified analyses showed that depressive symptoms of participants who were treated by mental healthcare nurses in Amsterdam (Wald = 31.360, df = 8, p = 0.000) or West-Friesland (Wald = 16.333, df = 8, p = 0.038) declined, while participants treated by the home care nurses showed no improvement of their depressive symptoms (Wald = 9.887, df = 8, p = 0.273).

Data from the qualitative interviews could shed light on this finding. Factors related to healthcare professions that emerged from the data included: their backgrounds, and interactions between different healthcare professionals.

### Healthcare professionals’ backgrounds

Nurses’ differing backgrounds and skills appeared to be of great influence on their attitudes toward the programme, confidence in their capacities, and presumably the way they provided the interventions.

Home care nurses were used to working with clients with mainly physical disabilities, in a solution-based manner that led to quick, visible results, and felt that the ‘Lust for Life’ education did not provide them with sufficient equipment to provide the interventions. Perhaps as a consequence, they perceived the ‘Lust for Life’ work as burdening and doubted the suitability and efficacy of the clinical interventions since they perceived little change in people’s moods (see Additional file 2: Box 5, quote 12). They further questioned the eligibility of participants for the programme because it took much effort to motivate them to accept and work with the interventions (see Additional file 2: Box 5, quote 13).

On the other hand, nurses of the mental healthcare organisations were (very) experienced in working with older people with depressive symptoms. They perceived the training as not challenging enough, were confident about their own skills to provide the interventions, were supportive of the interventions, and perceived the limited motivation of elderly people as a significant challenge in their jobs instead of as a characteristic of their clients (see Additional file 2: Box 5, quote 14).

### Healthcare professionals’ interactions

The nurses and physiotherapists did not experience working together as a team, and they missed out on information and involvement from one another. More precisely, physiotherapists would have liked to receive more information about the intake from the nurses while the nurses regretted the very limited involvement of the GPs since they all saw the advantages of more contact with them. Most GPs however did not mention concerns about not knowing the “Lust for Life” nurses. Perhaps this is related to the fact that GPs were mostly motivated for the programme because of the case finding procedure, and not necessarily because of the interventions that followed. An important factor that facilitated professional’s interactions in West-Friesland (compared to the other locations) was that nurses and physiotherapists were able to work in the same electronic medical file, which enhanced the exchange of information.

### Factors related to the innovation (*i.e*., the clinical interventions)

About the relationship between intervention choice and effectiveness, the most important facilitating factor that emerged from interviews with almost all participants was the interaction and personal contact with the nurses and/or physiotherapists. About the suitability of the interventions and self-perceived effects, participants and nurses were most critical about the guided self-help course, and nurses had doubts about the effects of Life Review and PST. About the provision of the clinical interventions, the limited embedding of the programme in different organisations and healthcare professions was seen as the most important limiting factor. See Additional file 3 for the detailed results.

### Context-related factors

The limited embedding of the programme in different organisations and healthcare professions put pressure on especially the home care nurses. Their high workload often kept them from spending sufficient time on training and providing the interventions (see Additional file 2: Box 7, quote 22).

It also had consequences for the way participants with persistent depressive symptoms were referred to additional treatments after having finished step two. Referrals to a general practitioner were scarcely made for participants treated by home care nurses and nurses of a mental healthcare organisation aimed at short-term treatment. However, for nurses of specialised mental healthcare organisations, transfers to step three were more easily made. They frequently transferred the participants they treated in the ‘Lust for Life’ program to their regular caseloads when symptoms persisted (see Additional file 2: Box 7, quote 23).

## Discussion

The aim of this study was to provide a contextualised understanding of factors facilitating and hindering implementation of the ‘Lust for Life’ programme. The programme consisted of two essential elements: first, we performed proactive case-finding, and second, provided stepped care preference-led interventions by offering multiple treatment options in every step. Our study showed a) that large-scale proactive case finding through screening to recruit participants was ineffective. Yet, the clinical interventions themselves b) reduced depressive symptoms. Our lessons were that the agreement between participant and healthcare professional on perceived need for care and type of intervention were crucial. Our data suggest that one single preference-led intervention is perhaps as effective as a whole programme.

### Stepped care preference-led interventions

To optimize treatment of late life depressive symptoms, we offered evidence-based interventions at the lowest possible intensity (stepped care) and provided interventions according to respondents’ preferences (preference-led care). Drop-out rates were still considerable (see Figure 4) [33]. We advise to take into account perceived needs for help. Interventions aimed at personal contact with others or with the nurses suited people’s needs better than the (more individually) guided self-help course. Participants were most enthusiastic about the exercise programme or life review. Several interviewees considered PST to be too difficult. The higher drop-out for the PST users compared to the life review group confirmed this limitation. Remarkably, the program was only effective in the first three months, and many people decided against second or third steps when indicated.

To explore implementation potential for other healthcare workers less used to working with older people with emotional distress, both (experienced) mental healthcare nurses and home care nurses offered the clinical interventions to explore implementation potential for other healthcare workers less used to working with older people with emotional distress. Older people treated by a mental healthcare nurse had fewer depressive symptoms, whereas persons treated by home care nurses showed no improvement. These results should be interpreted with care since allocation to mental healthcare or home care nurses was not randomised.

### Strengths and limitations

Strengths are the use of both quantitative and qualitative methods that enabled us to gain a comprehensive and contextualised understanding of the implementation process. Data triangulation further strengthens our conclusions since findings obtained by qualitative and quantitative data point into the same direction. Another strength concerns our extensive qualitative research with all stakeholders involved in the implementation process, and our thorough analyses of the data by having two researchers double-code and discuss all the data collected. Lastly, the chosen stepped-wedge randomised-cluster design gave us a unique opportunity to study the implementation process and consequently change and study the (adjusted) implementation strategy in following clusters according to significant findings.

Our study also has limitations. A stepped wedge design is attractive for implementation research as it allows both an estimate of effect and a flexible, hybrid design, making it possible to improve implementation. However, this type of design has more threats to the validity of the comparisons of the different interventions than, for instance, cluster randomised trials, including the non-randomised allocation and power issues. Second, during interviews and focus groups, we could have focused more on facilitating factors. Context-related factors were also slightly underrepresented in our data. Third, to prevent drop-out and adjust the programme to participants’ preferences, we provided an opportunity to choose. Fortunately, the impact of this protocol deviation was limited since only five persons (4%) chose. Yet, since data from quantitative and qualitative findings and perspectives from multiple stakeholders all point to the same relevant factors, we feel that we have captured the most important perspectives on implementation.

## Conclusions

Although the ‘Lust for Life’ programme reduced depressive symptoms, several factors on all implementation levels hindered implementation. Our findings add to the notion that universal prevention by depression programmes, with a screening component addressing an entire population, is not effective and should not be rolled out in daily practice. This is an important finding in the Dutch context of insurers gradually making screening procedures mandatory in primary care [34]. Before treatment modalities are to be provided, we also plead for significant attention to illness perceptions and perceived need for care. Perhaps it suffices to offer all available interventions at once instead of providing them according to stepped care principles. Adequate provision also requires that nurses have significant skills. Implementation is more suitable in (generic) mental healthcare settings or primary care than in home care organizations. The ‘Lust for Life’ program provides a good starting point for improving late life depression management in the community.

## Additional files

Additional file 1:
**Description of a stepped-wedge randomised-cluster design.**
Additional file 2:
**Quotations.**
Additional file 3:
**Additional results.**
