# Supplementary material for: Implementing an outreaching, preference-led stepped care intervention programme to reduce late life depressive symptoms: results of a mixed-methods study
Source: Implement Sci. 2014 Aug 28;9:107. doi: 10.1186/s13012-014-0107-y (PMC4156632; doi:10.1186/s13012-014-0107-y)
Supplement: Additional file 1: — Description of a stepped-wedge randomised-cluster design. [file 13012_2014_107_MOESM1_ESM.docx]

Box 1: Description of a stepped-wedge randomised-cluster design

| A stepped-wedge design is a type of crossover design in which the intervention is rolled out sequentially to participants over a number of time periods. All participants are recruited at the start of the study and assigned to several clusters. Starting moments are determined by (cluster-) randomisation and by the end of the study, all participants will have received the intervention. That is, clusters cross over from control to intervention condition. During the control condition, usual care is provided. |
| --- |
