# Supplementary material for: Implementing an outreaching, preference-led stepped care intervention programme to reduce late life depressive symptoms: results of a mixed-methods study
Source: Implement Sci. 2014 Aug 28;9:107. doi: 10.1186/s13012-014-0107-y (PMC4156632; doi:10.1186/s13012-014-0107-y)
Supplement: Additional file 2: — Quotations. [file 13012_2014_107_MOESM2_ESM.docx]

| *Box 1: Pro-active case finding: factors related to participants with depressive symptoms* |
| --- |
| Quote 1  “*Yes, once in a while* [I have blue moments] *but that happens to everybody […] But erm, nothing to speak of.”* (resp#11, a 67-year old female).  Quote 2  *“If those things occur* [physical problems]*, then I’m not in a very good mood. And there is nothing you can do about it.”* (resp#16, an 88 year-old female).  Quote 3  “*Lots of people who experience it as a status quo, or as something that belongs to them or something that has always been this way.”* (nurse, MHC 1). |

| *Box 2: Pro-active case finding: factors related to health care professionals (and their interactions)* |
| --- |
| Quote 4  *“Those people who think ‘oh, it is not that bad’ or ‘no, I don’t have mental but physical complaints’. I think that if the general practitioner does not refer them, you cannot catch these people.”* (nurse, MHC2). |

| *Box 3: Pro-active case finding: factors related to* the innovation |
| --- |
| Quote 5  “*There were people who were totally shocked, who wanted to unsubscribe, because I enlisted them and they did not feel depressed. I found that irritating.”* (GP#4).  Quote 6  *“So I have no idea what it means and what the result will be. And I feel: Let’s wait and see.”* (resp#27, a 71 year-old female).  Quote 7  “*When you came up with this project, I had a very different group in mind. They all said ‘no’ in the end. […] The people that visit my surgery regularly with symptoms of anxiety and depression […] the lonely people.”*(GP#2). |

| *Box 4: The preference-led, stepped care interventions: Factors related to the participants with depressive symptoms* |
| --- |
| Quote 8  *“And some people know straight away: mostly the physical exercise people who almost immediately say: ‘oh no, physical exercise’, that’s what they say.”* (HC-nurse)*.*  Quote 9  *“Well, because I have always liked to be physically active in life. […] So the choice was not difficult at that moment.”* (resp#17, an 83 year-old male).  Quote 10  *“Well yes, because I had the feeling I still had a long way to go. So if there is more… I really want to do everything to get rid of that.”* (Resp#31, a 72 year-old female).  Quote 11  *“I say: boy, it is okay for you to come visit me […] That is not the point. […] I doesn’t bring me anything, and I think it is a waste […] of your time, of my time. […] And there are many things like that I will deal with myself.”* (resp#13, an 88 year-old female). |

| *Box 5: The preference-led, stepped care interventions: Factors related to the health care professionals (and their interactions)* |
| --- |
| Quote 12  “*We see now how much time it costs* [to conduct the programme*] but most of all how much psychological burden [*it puts on us; R3]*. […] It is quite a different role we have [in this program] […] our* [usual] *work is more goal-oriented to put it like that. Then there are problems, and you can help to solve those problems* [R2]*.” “I am really struggling […] It takes so much effort and I keep on trying but it takes so much energy from me, and I just do not see any results. I do not see any progress at all* [with my clients; R1]*”.*  *Quote 13*  *“*[R1 to R2] *Yes, I understand you just feel that those people are not be motivated.* [R2: yes]*. Well, that’s more or less what I think too.”* (nurses HC).  Quote 14  *“I really have spent an hour sitting there, and when it was necessary I went back again […]. The aim is that people […] make a decision based on full information and trust. And when you feel that that is not there yet- you just have to go on a bit, I think.”* (nurse MHC2). |

| *Box 6: The preference-led, stepped care interventions: Factors related to the innovation (i.e. the clinical interventions)* |
| --- |
| Quote 15  *“Well, she asks how I’m doing. And she also gives nice advice. She is very dedicated.”* (resp#31, a 72 year-old female).  Quote 16 (about Life Review)  *“Because I have noticed several times that people have a need to talk about the past and about their memories.”* (nurse MHC1)*.*  Quote 17  *“You talk to one another. […] I find it really nice that I have someone to talk with here in my own home.”* (resp#6, a 77 year-old male).  Quote 18  *“They all like it immensely that we pay them so much attention, that we visit. […] That is what they mention at the end […] What a pity that you won’t come here anymore, that’s what it’s about. […] But not about what they are doing.”* (nurse HC)*.*  *Quote 19*  *“When people are still ‘all there’, alert, read a lot and are interested, well yes, then they make use of such a book and really do something with it. But I think very few people really understand what it is about.”* (nurse MHC2).  Quote 20  *“They don’t take it in when you are not sitting next to them. That really is my experience with all the people who have started with this book.”* (nurse HC)*.*  *Quote 21*  *“You want to teach them a method so that when there is no professional care, they can do something with it. Sometimes I have doubts about that, whether […] that is what they really need. […] You are working on those problems, you know, more than on teaching that method.”* (nurse MHC2). |

| *Box 7: The preference-led, stepped care interventions: Context-related factors* |
| --- |
| Quote 22  *“People have to do it in between […] And the fact that they participate in research that was approved by their employer, but at the same time they have to work their regular case load. Well, it’s just not possible. […] That’s why nurses sometimes find themselves in an impossible situation.”* (trainer).  Quote 23  *“To be honest, I just kept them. Well, they were already clients in our Mental Health Care Organization.”* (nurse MHC2). |
