# Supplementary material for: Implementing an outreaching, preference-led stepped care intervention programme to reduce late life depressive symptoms: results of a mixed-methods study
Source: Implement Sci. 2014 Aug 28;9:107. doi: 10.1186/s13012-014-0107-y (PMC4156632; doi:10.1186/s13012-014-0107-y)
Supplement: Additional file 3: — Additional results. [file 13012_2014_107_MOESM3_ESM.docx]

Additional results

Factors related to the innovation (i.e. the clinical interventions)

Many factors emerged at this level: 1) the relationship between intervention choice and effectiveness of the ‘Lust for Life’ program; 2) the suitability of the interventions and self-perceived effects; and 3) provision of the clinical interventions.

*Relationship between intervention choice and the effectiveness of the ‘Lust for Life’ program*

We performed several quantitative ancillary analyses to compare the relationship between the various intervention options that older people chose and persons’ course of depressive symptoms. The course of depressive symptoms was similar for people who participated in the self-help course versus the exercise programme (Wald=11.549, df=8, p=0.177) and for those who chose PST versus life review (Wald=13.658, df=8, p=0.091).

Second, the relationship between intervention modality and drop-out was analysed. There were no significant differences in drop-out rates between participants who took part in the self-help course and those who participated in the exercise program (OR=1.755, 95% confidence interval=0.801-3.846). We did however find that individuals who participated in life review had more chances of completing this intervention than persons who participated in PST (OR=4.714, 95% confidence interval=1.178-18.861). Results from the qualitative interviews could provide a more contextualised understanding of this finding.

*Suitability of the interventions and self-perceived effects*

The most important facilitating factor that emerged from interviews with almost all participants was the interaction with the nurses and/or physiotherapists. Irrespective of which interventions was chosen, participants placed high (some even most) value on their interaction with these professionals (see Box 6, quote 15).

It appeared that interventions that included a significant amount of personal contact with the nurse (i.e. life review) or with other elderly people (i.e. the exercise program) were highly appreciated by participants. Nurses noticed that these interventions best suited many elderly people’s needs (see Box 6, quote 16). Participants and physiotherapists were very enthusiastic about the group interactions during the exercise programme. Participants hardly expressed any criticism about these interventions and were positive about the results. People who participated in PST were most enthusiastic about their contact with the nurse, but perceived PST as a difficult intervention to understand and put into practice (see Box 6, quote 17). This was also seen by the nurses and by the trainer. On the other hand, nurses valued that PST could easily be learned by professionals and that it provides good opportunities to adjust to participants’ needs since participants are given the choice which problems they want to work on.

Participants and nurses were most critical about the guided self-help course, the only intervention that individuals had to work through more or less by themselves and that was not focused on interpersonal contact. Although this course was positively received by some participants, for most of them it did not match their needs. All teams of nurses had the impression that elderly people were more focused on their contact with the nurse than interested in the self-help course itself (see Box 6, quote 18). Other problems mentioned by participants as well as nurses were that it was difficult to find a way to adjust to participants’ needs when they did not perceive burdensome depressive symptoms or experienced very specific problems, and that the self-help course was too difficult for many participants to understand, to work with and to put into practice (see Box 6, quote 19). Despite these barriers, nurses generally valued the content of the book and the opportunity to choose from several exercises and information to adjust the course to participants’ individual situation and needs.

*Provision of the clinical interventions*

Irrespective of which intervention was chosen, participants highly valued the possibility to receive the nurse at home for the intake session and (most of) the interventions, instead of having to leave the house. Health care professionals mentioned several intervention-specific barriers to adequate provision of the interventions, which all concerned impossibilities to follow the protocol.

*Step one interventions: self-help course and exercise programme*

For the guided self-help course, this meant that although the course was developed as an intervention that required minimal guidance from a health care professional, nurses from all sites felt they were forced to take on an active role in providing the intervention by making frequent visits, going through the exercises together etcetera (see Box 6, quote 20). Nurses provided guidance at an average of 4 visits (sd=2) and 2.5 phone calls (sd=1.5) to each participant for clarification and encouragement.

For the exercise therapy, physiotherapists mentioned that the use of heart rate monitors to determine the exercise level at 60% of the maximum heart beat was impractical and intervened with an adequate provision of the exercises. Furthermore, it appeared to be very difficult to arrange a group of four to six participants to follow the exercise programme at the same time. Lastly, the physiotherapists in Amsterdam noticed that although participants were all dealing with depressive symptoms, these hardly ever came up for discussion but could have a big influence on the group process. They therefore experimented in cluster four with a short group conversation preceding some exercise sessions for participants to tell the others how they were doing, and found that this improved participants’ feelings of togetherness, concern for each other and motivation to do the exercises.

*Step two interventions: Life review and PST*

Nurses had their doubts about the effectivity of the step two interventions. Both interventions are aimed at teaching participants a certain method they can apply by themselves in the future to alleviate their mood, i.e. they are taught how to retrieve specific, positive memories from the past (life review) or how to solve daily problems (PST). Nurses questioned to which extent participants were actually capable of adopting these methods and therefore whether these interventions are effective in a long term. A nurse explained about PST (see Box 6, quote 21).

*Step three intervention: referral to general practitioner*

Originally, step three consisted of a referral to the general practitioner to discuss further treatment in specialised mental health care. Several nurses mentioned that, in their opinion, treatment in specialised mental health care was not indicated for many participants with mild persistent symptoms. Therefore, we changed the protocol early on. Also, additional costs were believed to retain many elderly people from accepting a referral. Therefore, general practitioners were advised to discuss a referral to specialised mental health care for participants with persistent depressive symptoms (PHQ > 6) and a depressive disorder at baseline. For others, other treatment options could also be considered such as a referral to the practice nurse, a psychologist, (preventive) group therapy etcetera.
